# Cellular and Molecular Mechanisms in the Pathogenesis of Classical, Vascular, and Hypermobile Ehlers‒Danlos Syndromes

**DOI:** 10.3390/genes10080609

**Published:** 2019-08-12

**Authors:** Nicola Chiarelli, Marco Ritelli, Nicoletta Zoppi, Marina Colombi

**Affiliations:** Division of Biology and Genetics, Department of Molecular and Translational Medicine, University of Brescia, 25121 Brescia, Italy

**Keywords:** autophagy, collagen III, collagen V, Ehlers‒Danlos syndrome, endoplasmic reticulum, extracellular matrix, fibroblast-to-myofibroblast transition, miRNA, transcriptome, wound healing

## Abstract

The Ehlers‒Danlos syndromes (EDS) constitute a heterogenous group of connective tissue disorders characterized by joint hypermobility, skin abnormalities, and vascular fragility. The latest nosology recognizes 13 types caused by pathogenic variants in genes encoding collagens and other molecules involved in collagen processing and extracellular matrix (ECM) biology. Classical (cEDS), vascular (vEDS), and hypermobile (hEDS) EDS are the most frequent types. cEDS and vEDS are caused respectively by defects in collagen V and collagen III, whereas the molecular basis of hEDS is unknown. For these disorders, the molecular pathology remains poorly studied. Herein, we review, expand, and compare our previous transcriptome and protein studies on dermal fibroblasts from cEDS, vEDS, and hEDS patients, offering insights and perspectives in their molecular mechanisms. These cells, though sharing a pathological ECM remodeling, show differences in the underlying pathomechanisms. In cEDS and vEDS fibroblasts, key processes such as collagen biosynthesis/processing, protein folding quality control, endoplasmic reticulum homeostasis, autophagy, and wound healing are perturbed. In hEDS cells, gene expression changes related to cell-matrix interactions, inflammatory/pain responses, and acquisition of an in vitro pro-inflammatory myofibroblast-like phenotype may contribute to the complex pathogenesis of the disorder. Finally, emerging findings from miRNA profiling of hEDS fibroblasts are discussed to add some novel biological aspects about hEDS etiopathogenesis.

## 1. The Extracellular Matrix: An Overview

Connective tissues have an extracellular matrix (ECM) with a specific composition generated during embryogenesis and maintained in adult life. The ECM is a complex network that provides a structural scaffold to the surrounding cells and is a reservoir of bioactive molecules such as cytokines and growth factors that control cell behavior [1]. The main ECM components include proteoglycans, hyaluronic acid, adhesive glycoproteins such as fibronectin and laminins, and fibrous proteins like collagens and elastin [2]. Matricellular proteins such as thrombospondins, osteopontin, periostin, and tenascins are non-structural ECM proteins, primarily acting as mediators of cell–matrix interactions, which are abundantly expressed during embryonic development, wound healing, and tissues renewal [3].

The human matrisome consists of about 300 macromolecules comprising the “core matrisome”, which is composed of many different collagens, proteoglycans (e.g., aggrecan, versican, perlecan, and decorin), and glycoproteins (e.g., laminins, elastin, fibronectin, thrombospondins, and tenascins) [4]. Matrisome also includes many matrisome-associated proteins and ECM-regulators, i.e., ECM-cross-linking (e.g., lysyl oxidases, transglutaminases) and ECM-modifying enzymes (e.g., proteases and their inhibitors) together with secreted factors including transforming growth factor β (TGFβ), wingless integrated (Wnt), and multiple cytokines [5].

Collagens represent the major ECM structural components and play a central role in providing the structural integrity of several connective tissues (e.g., cartilage and bone) and various organ systems including skin, lungs, blood vessels, and cornea. Collagens are also involved in cell adhesion, chemotaxis, and migration [1,2]. The dynamic interplay between cells and collagens regulates tissue remodeling during growth, differentiation, morphogenesis, and wound healing [6,7]. The common molecular feature of collagens is their triple helical structure, which consists of three collagenous α-chains with the typical recurring (Gly-Xaa-Yaa)_n_ tripeptide sequence. The presence of glycine residues in the collagenous domain is essential for stability and correct assembly of the triple helix. Collagen biosynthesis, assembly, and maturation require a sequence of well-controlled intracellular and extracellular events (for review see [8,9]). Collagen I is the most abundant type expressed in bone, cornea, dermis, and tendon. Collagen III is primarily present in the tunica media of the blood vessels and hollow organs (e.g., uterus, intestine). Collagen V is widely distributed, especially in dermis, tendons, and muscles, playing a central role in collagen I fibrillogenesis [9]. These fibrillar collagens form structures necessary to ensure the strength and structural integrity of the ECM of all connective tissues and organs of the body [10].

Fibronectin is a dimeric and fibrillar glycoprotein ubiquitously organized in the ECM of all tissues and is also present in soluble form in the plasma. Cellular fibronectin self-assembles in fibers and binds collagens, fibrin, proteoglycans, and cell surface receptors, providing cell growth, adhesion, and migration. During wound healing, it forms a provisional matrix with fibrin and enters in the granulation tissue formation in the late phase of re-epithelization [11].

In addition to ensuring physical support and structural integrity, the proper ECM composition and organization are crucial for cell health. ECM undergoes a continuous turnover either under physiological or in pathological circumstances, and its homeostasis is critical for connective tissues architecture and function [12,13]. Integrins are specific cell surface receptors that mediate the complex cell-matrix interactions. These bridging molecules, which are heterodimeric transmembrane receptors containing α and β subunits, connect ECM to cytoskeleton by interacting via their extracellular domain with collagens and other matrix molecules and via their cytoplasmic tails with cytoskeleton components (e.g., actin, vinculin, talin, paxillin), thus mediating cell adhesion and motility [14,15].

## 2. Pathological ECM Remodeling and Perturbation of Cellular Homeostasis

Cell-matrix interaction via integrins is crucial for cell survival and tissue homeostasis. Prolonged loss of integrin-mediated cell–ECM adhesion leads to anoikis [16]. Under physiological conditions, ECM detachment triggers anti-apoptotic signals as a cell survival mechanism to delay the onset of anoikis. One of such signaling pathways is autophagy, which is a highly conserved cellular catabolic process that promotes homeostasis and mitigates the stress due to ECM detachment [17]. Autophagy is essential for cellular maintenance and homeostasis by promoting the turnover of macromolecules and organelles via the lysosomal degradative pathway [18]. Physiological and pathological changes in the ECM composition play a crucial role in modulating autophagy activity [19,20]. For instance, deficiency of collagen VI, which is associated with a spectrum of different myopathic conditions, perturbs ECM architecture, impairs the autophagic flux, and activates pro-apoptotic signals [21]. Autophagy, in turn, contributes to the maintenance of endoplasmic reticulum (ER) function by mediating its turnover through the autophagic sequestration of ER fragments into autophagosomes, the so-called ER-phagy process [22].

ECM components also modulate immune cell migration into inflamed tissues and their activation and proliferation [23]. The stimulation of the innate immunity results from the recognition of specific mediators, namely pattern recognition receptors, which, in turn, recognize molecules, referred as danger-associated molecular patterns, which are released from damaged tissues [24]. It is well documented that different ECM components or their fragments including the fibronectin 1 extra domain A, one of the alternative spliced regions of fibronectin encoding gene, tenascin-C, fibrinogen, and several proteoglycans, serve as danger signals and trigger immune responses following tissue damage or in response to pathological ECM remodeling [24,25]. In fibrotic conditions, increased ECM production, accumulation of ECM fragments, augmented secretion of cytokines, fibroblast-to-myofibroblast transition, and activation of immune responses, dependent on toll-like receptors, occur [26]. The regulation of ECM synthesis and remodeling is central for human health, as recognized in different heritable connective tissue disorders [1,27]. Indeed, molecular defects in a large range of ECM-related genes, including those encoding enzymes involved in biosynthesis or processing of ECM proteins, cause a myriad of connective tissue disorders, e.g., Ehlers–Danlos syndromes, Osteogenesis imperfecta, Marfan syndrome, Loeys–Dietz syndromes, arterial tortuosity syndrome, and numerous skeletal dysplasias [1,27]. These disorders are characterized by a multisystem involvement in terms of cardiovascular, skeletal, and cutaneous features [28], highlighting the functional relevance of the ECM in ensuring the integrity and function of several connective tissues.

The pathological consequences of defects in ECM components depend on the balance between extracellular effects, e.g., reduced protein secretion and export of misfolded proteins, and intracellular consequences such as apoptosis activation, ER dysfunction, and autophagy perturbation that impact in different ways on the molecular pathology and disease severity [27,29,30,31].

## 3. Ehlers‒Danlos Syndromes

Ehlers‒Danlos syndromes (EDS) represent a clinically and genetically heterogeneous group of conditions that share a variable combination of skin hyperextensibility, joint hypermobility, and internal organ and vessel fragility [32]. The 2017 international classification of the Ehlers‒Danlos syndromes recognizes 13 subtypes, which are caused by pathogenic variants in 19 different genes, mainly encoding fibrillar collagens and collagens-modifying proteins [32]. EDS types are grouped based on the underlying genetic and pathogenetic mechanisms in disorders related to (i) collagens primary structure and processing (*COL1A1*, *COL1A2*, *COL3A1*, *COL5A1*, *COL5A2* and *ADAMTS2*), (ii) collagens folding and cross-linking (*PLOD1* and *FKBP14*), (iii) structure and function of the myomatrix, i.e., the specialized ECM of muscle (*TNXB* and *COL12A1*), (iv) glycosaminoglycans biosynthesis (*B4GALT7*, *B3GALT6*, *CHST14*, and *DSE*), (v) complement pathway (*C1S* and *C1R*), and (vi) intracellular processes (*SLC39A13*, *ZNF469*, and *PRDM5*). The classical (cEDS), vascular (vEDS) and the molecularly unsolved hypermobile (hEDS) EDS forms account for more than 90% of patients. Recently, a new and very rare EDS variant has been identified that is caused by biallelic mutations in the *AEBP1* gene (Table 1) [33,34,35,36].

The new nosology proposed for each subtype a set of major, minor, and minimal criteria addressing clinical suspicion for a specific EDS type and confirmatory molecular testing. For a comprehensive overview of all EDS forms see the landmark work by Malfait and colleagues [32].

The decrease in the tensile strength and integrity of skin, joints, and hollow organs is a common disease mechanism shared by the different EDS types [37]. This mechanical weakness is considered the driving factor of connective tissue fragility, even if it is likely that multiple cell-matrix interplays and involvement of distinct intracellular signaling pathways contribute to the molecular pathology of the different EDS phenotypes [38].

In the following chapters, we will review and expand the results derived from our previous transcriptome and in vitro studies on cEDS, vEDS, and hEDS patients’ dermal fibroblasts. Taken together, these studies highlighted that the alteration of the ECM structural integrity is a common disease factor contributing to the pathogenesis of all these conditions.

In cEDS and vEDS fibroblasts, the ECM disarray is a direct consequence of molecular defects in respectively collagen V and collagen III that impair common molecular functions essential to guarantee adequate folding and maturation of proteins and biological processes crucial for cell survival and homeostasis. The ECM disorganization observed in hEDS cells may be a consequence of an excessive pathological turnover, mainly due to ECM-degrading enzymes and other so far unknown factors, which might be primary contributors involved in the transition to a pro-inflammatory myofibroblast-like phenotype. Consistently, the perturbation of distinct transcriptional patterns observed in cEDS, vEDS, and hEDS fibroblasts pointed out different disease mechanisms underlying the pathophysiology of these EDS cell types.

Altogether, these insights represent a starting point for future investigations on the numerous pathobiological aspects underlying these conditions. An overview of the biological findings emerged from transcriptome and in vitro studies on dermal fibroblasts from cEDS, vEDS, and hEDS patients is summarized in Table 2.

## 4. Classical Ehlers‒Danlos Syndrome

Classical EDS (cEDS, OMIM#130000) is characterized by marked skin involvement, generalized joint hypermobility, and abnormal wound healing [32,39]. Most patients harbor point mutations or chromosomal rearrangements in *COL5A1* or *COL5A2* genes encoding the collagen V [40,41]. This collagen is abundantly distributed in a variety of tissues as heterotrimers, which co-assemble with collagen I to form heterotypic fibrils [42].

Collagen V knockout mice synthesize and secrete normal amounts of collagen I, but collagen fibrils are absent, and the animals die at the onset of organogenesis, supporting the crucial role of collagen V for embryonic development [43].

Collagen V haploinsufficiency is the most common molecular defect caused by *COL5A1* null alleles, whereas rare *COL5A1* variants and the majority of *COL5A2* mutations reported so far affect collagen V structural integrity by exerting a dominant negative effect [40,41].

## 5. Altered ECM Turnover, Wound Healing, and Inflammation in cEDS Fibroblasts

Although the reduced availability of collagen V is crucial in the pathogenesis of cEDS, the molecular aspects contributing to the pathophysiology of the disorder remain poorly characterized. Our in vitro findings demonstrated that cEDS patients’ fibroblasts show disassembly of many ECM components, including collagen V and III, fibronectin, and fibrillins, and disorganization of collagen- and fibronectin-specific α2β1 and α5β1 integrin receptors [44,45,46,47]. cEDS cells also exhibit a reduced in vitro migration capability, an abnormal wound healing response, and a crosstalk involving the αvβ3 integrin and epidermal growth factor (EGF) receptor that rescues them from anoikis [44,45,46,47,48,49,50]. In line with these in vitro findings, *Col5a1* and *Col5a2* deficient mice show a defective wound healing response and reduced cell migration [51,52].

Transcriptome profiling of cEDS fibroblasts added new insights into the complex molecular mechanisms involved in the maintenance of ECM homeostasis and proper wound healing, since patients’ cells showed the dysregulated expression of many genes encoding matricellular and soluble proteins with prominent functions in cell proliferation and migration, collagen assembly and ECM remodeling during wound healing, i.e., *SPP1*, *POSTN*, *EDIL3*, *IGFBP2*, and *C3* [47]. Wound healing is a highly controlled multistep process involving several growth factors, cytokines, matrix metalloproteases, and cellular receptors, as well as proper crosstalk of different ECM constituents essential for ensuring tissue regeneration [53]. In addition to ECM glycoproteins and collagens, many other matricellular proteins including osteopontin, periostin, and tenascins are required for the formation of a provisional ECM during wound repair [54,55].

Osteopontin encoded by *SPP1,* which shows a decreased expression in patients’ cells, is involved in several physiological processes related to inflammation, biomineralization, cell viability, and wound healing [56,57]. Through its interaction with the αvβ3 integrin, osteopontin facilitates the adhesion of bone cells during bone tissue formation by stimulating a mineralized collagen ECM [58,59]. Finally, the functional role of osteopontin in the ECM reorganization during wound healing is crucial, since *Opn*-deficient mice show ECM disorganization and disassembly of collagen fibrils in the deep layers of wound sites [60].

In cEDS fibroblasts, the decreased expression of periostin encoded by *POSTN* may also contribute to the generalized ECM disarray and in vitro poor wound healing [44,48]. Indeed, periostin plays an important role in ECM structure and organization and particularly in collagen assembly, by acting as a scaffold protein for the bone morphogenetic protein 1, which facilities the proteolytic activation of lysyl oxidase that, in turn, catalyzes the covalent cross-link formation of collagens [61]. Consistently, *Postn*-deficient mice exhibit marked reduction of collagen cross-linking and increased levels of collagen fragments owing to proteolytic digestion [62,63]. Periostin also acts as a pro-survival protein in many cellular circumstances by interacting with αvβ3 and αvβ5 integrin receptors and mediating the activation of several intracellular signaling pathways [64]. In wound sites, it promotes activation of fibroblasts during wound contraction and stimulates collagen assembly and ECM reorganization [65,66]. *POSTN* not only shows a diminished expression in vEDS cells but also in dermal fibroblasts from patients with *FKBP14*-kEDS [67], further emphasizing the crucial role of periostin as a scaffold matricellular protein necessary for collagen assembly and ECM stability.

*EDIL3* (EGF-like repeat- and discoidin I-like domain-containing protein 3), the most down-regulated transcript in cEDS fibroblasts, encodes an ECM-associated protein that promotes angiogenesis in vitro through binding to αvβ3 and αvβ5 integrins [68]. It stimulates cell migration and proliferation, mediates apoptotic cell phagocytosis, regulates neutrophil recruitment to the inflamed tissue, and prevents chondrocyte anoikis through its interaction with the αvβ3 integrin [69,70,71].

*IGFBP2* (insulin-like growth factor-binding protein 2), the most up-regulated gene in cEDS cells, enhances cell migration in different cell types through its binding to αvβ3 and α5β1 integrins [72,73]. Its high expression in cEDS cells might represent a transcriptional response in the attempt to counteract, at least in vitro, their reduced migration capability [48,49].

Of note is also the marked up-regulation of the complement factor C3 belonging to a complex network of plasma and membrane proteins involved in the innate immunity [74]. Complement can modulate the inflammatory response during wound healing to restore tissue injury; however, its unbalanced or prolonged activation can exacerbate inflammation, delaying the physiological wound healing [75]. Specifically, C3 functions as a negative regulator of tissue healing, since *C3*-deficient mice exhibit an increased wound healing and angiogenesis [76].

Taken together, these gene expression abnormalities expand the current understanding of altered molecular mechanisms underlying the deficient wound healing response observed in cEDS cells. Additional functional work might help to establish the concrete involvement of these proteins including the αvβ3 integrin in the impaired wound healing, which likely leads to the cutaneous manifestations of cEDS [39,77].

## 6. Perturbation of ER Homeostasis and Autophagy in cEDS Fibroblasts

ER is a fundamental cellular organelle involved in the maintenance of numerous aspects of cell health by ensuring folding and exporting of secretory or transmembrane proteins [78]. The biosynthesis, processing, and integrity of collagens and other ECM structural constituents are critical for intracellular proteostasis [79]. To restore intracellular equilibrium, the ER counteracts the accumulation of aggregated or misfolded proteins by means of quality control mechanisms such as unfolded protein response, ER-associated degradation, and autophagy [80].

In cEDS fibroblasts, a possible unbalance of ER homeostasis and autophagy was assumed given the decreased expression of many associated genes such as *DNAJB7*, *ATG10*, *CCPG1*, and *SVIP* [47]. *DNAJB7* encodes a member of the J protein/heat shock protein family acting as ER chaperones in the quality control of aggregate protein [81]. *ATG10* is a member of the autophagy related proteins family that participates to the generation and expansion of autophagosomes [22]. The protein encoded by *CCPG1* acts as an ER-phagy cargo receptor facilitating the attachment to growing autophagosomes of the microtubule-associated LC3 protein that is crucial for autophagosome maturation [22,78,82].

CCPG1 plays a key role in the ER proteostasis, since its deficiency causes accumulated insoluble proteins and consequent ER dilation [83]. A recent study reported the contribution of ER-phagy in the selective degradation of misfolded procollagen I molecules via a calnexin-FAM134B complex [84]. Furthermore, inefficient procollagen folding in the ER may induce autophagy as a cytoprotective mechanism [85]. Based on these findings, it is reasonable to speculate a possible role of CCPG1 in this autophagy-dependent mechanism and that its decreased expression in cEDS fibroblasts might impair the ER quality control. The disturbance of ER homeostasis in cEDS is also suggested by the decreased transcription of the small VCP/p97-interacting protein (SVIP), which is a modulator of the ER-associated degradation pathway [86,87]. Previous and recent data highlighted the contribution of SVIP in the regulation of autophagy, since its overexpression is associated with increased LC3 lipidation and attenuation of hepatic fibrosis by the induction of the autophagic flux [88,89].

The impairment of ECM organization, matrix-cell interactions, and the activation of ECM-dependent intracellular signaling may elicit autophagy [18,19]. Cell detachment from the ECM activates the autophagy pathway that, in turn, protects cells from anoikis [90]. Moreover, depletion of autophagy regulators is associated with induction of pro-apoptotic signals, decrease of collagen degradation via lysosome pathway, and regulation of cell adhesion [90,91]. In line with these observations, the aberrant expression of collagen V and defective remodeling of ECM in cEDS cells might affect ER homeostasis and autophagy, and consequently activate a pro-survival mechanism mediated by a crosstalk between αvβ3 integrin and EGF receptor [45,50]. Additional studies are needed to better elucidate the contribution of these processes in the molecular pathology of cEDS.

## 7. Vascular Ehlers‒Danlos Syndrome

Among the different EDS forms, vascular EDS (vEDS, OMIM#130050) is the most severe type and is primarily characterized by life-threatening features of tissue fragility leading to arterial dissection or aneurysm, gastrointestinal ruptures, and pregnancy complications at a young age [92,93].

vEDS is caused by mutations in *COL3A1* encoding collagen III that shows a predominant expression in blood vessels and hollow organs [94]. Most disease-causing variants in *COL3A1* are glycine substitutions that destroy the triple helical winding, thus altering the structural integrity of collagen III due to misfolded procollagen III in the ER, and thereby impairing the secretion and deposition into the ECM of functionally mature molecules [95,96].

*Col3a1* deficient mice show a reduced lifespan mainly due to arterial ruptures and abnormalities of collagen fibril organization in several collagen-rich organs, i.e., aorta, skin, lung, and bowel [10].

Our previous protein findings on cultured patients’ fibroblasts showed that dominant negative mutations in *COL3A1* lead to the reduced secretion of collagen I into the ECM [44], consistent with the known regulatory role of collagen III in synthesis and deposition of heterotypic fibrils that largely contain collagen I [96].

## 8. Disturbance of ECM Organization, Collagens Processing, and ER Homeostasis in vEDS Fibroblasts

Although it is well known that the disruption of the collagen III triple helical structure leads to abnormal protein folding, different biological aspects of the vEDS pathogenesis are not yet fully studied. In line with cEDS and hEDS transcriptome profiling, vEDS fibroblasts show the differential expression of several genes encoding structural constituents of the ECM, further supporting the notion that abnormal ECM remodeling is a common denominator of these conditions [50,97].

vEDS cells showed a marked decrease in expression of the fibrillin 2 encoding gene (*FBN2*). Fibrillins are essential structural ECM components involved in the organization of blood vessels and dermis, and in combination with elastic fibers they act as scaffold to ensure tissue elasticity [98]. Moreover, fibrillins interact with elastin microfibril interface-located proteins (EMILINs) and facilitate their incorporation into the dermal ECM [99].

Fibrillins also regulate the bioavailability of the TFGβ through the interaction with latent TFGβ binding proteins [99]. Specifically, fibrillin 2 plays a role in bone and soft connective tissue morphology by influencing the collagen cross-linking [100].

Besides its role in elastogenesis and ECM stability, fibrillin 2 also has a role during wound healing [101]. About this, vEDS fibroblasts share with cEDS cells the decreased expression of the related periostin-encoding gene and show reduced migration capability [49]. Consistently, both altered wound healing and reduced total collagen content were reported in a *Col3a1* transgenic mouse model [102].

Our protein findings confirmed the pathological ECM remodeling of vEDS fibroblasts, as a generalized fibrillin disarray in combination with the disassembly of EMILINs and elastin network was revealed, consistent with the extreme vascular fragility observed in vEDS patients [97]. The disorganization of core proteins of the proteoglycans perlecan, versican, and decorin, which are involved in the formation of collagen fibrils, further emphasizes the widespread ECM disarray and altered collagens biosynthesis/secretion of vEDS cells that are consequent to collagen III defect [97].

The biosynthetic pathway of fibrillar collagens is a highly regulated process involving folding enzymes, molecular chaperones, and post-translational modifications essential for proper protein assembly, stability of collagen fibrils, and their transport to the cell surface [8,9]. In vEDS cells, this complex machinery seems to be perturbed given the reduced expression of many ER-resident enzymes involved in different steps of collagen biosynthesis, i.e., *P4HA2, P4HA3, LOXL3,* and *FKBP14*.

*P4HA2* and *P4HA3* encode the α-subunit of the collagen prolyl-4-hydroxylase, which catalyzes the hydroxylation of collagen prolyl residues necessary to provide thermal stability to the collagen triple helix. Lysyl oxidase-like 3 (*LOXL3*) stabilizes the formation of intra- and intermolecular crosslinks during assembly of collagen and elastin fibrils. *FKBP14* encodes a peptidyl-prolyl cis-trans isomerase (FKBP22) that catalyzes in the ER lumen collagen folding and it functions as a molecular chaperone for different collagens including collagen III [8,103]. Dermal fibroblasts of *FKBP14*-deficient patients show a generalized perturbation of protein folding and a consequent enlargement of ER cisternae [104]. The marked decrease of FKBP22 protein levels observed in vEDS cells suggests an ER accumulation of misfolded proteins, consistent with the possible dilation of ER cisternae evinced by immunofluorescence analysis with the ER marker PDI [97].

Structural mutations in different collagen types disturb the assembly into hetero- or homotrimers or lead to abnormal triple helix folding. The consequent accumulation of misfolded collagen molecules into the ER lumen activates the proteasomal degradation system to re-establish ER proteostasis [27]. In vEDS cells, this quality control machinery might not work properly, given the decreased transcription of several genes encoding different catalytic and non-catalytic subunits of the proteasome complex, such as *PSMA6*, *PSMB6*, *PSMC3*, and *PSMD2*. In addition, the reduced transcription of members belonging to the DnaJ heat shock protein family, i.e., *DNAJB7*, *DNAJB11*, *DNAJC3, DNAJC10*, and *DNAJC24*, and to the thioredoxin superfamily, i.e., *TXN*, *PDIA4*, *PDIA5*, and *PDIA6*, which all act as intracellular mediators for correct protein folding and intracellular redox balance [105], further corroborates a perturbed ER proteostasis in vEDS fibroblasts.

This imbalance can be overcome by the activation of stress-related pathways, such as unfolded protein response and autophagy to restore basal cellular equilibrium [80]. The alteration of the ER redox state may also trigger pro-death signals through the regulation of members of the Bcl-2 family and activation of caspase-dependent apoptosis [106,107]. Consistently, we previously demonstrated that vEDS fibroblasts are in a pre-apoptotic state, due to downregulation of the Bcl-2 anti-apoptotic protein and increased levels of caspase enzymes, and activate a cell survival mechanism through an αvβ3-EGFR crosstalk [45,50]. However, in vEDS cells an enhanced expression of unfolded protein response-related genes was not identified, consistent with the recent findings on cultured dermal fibroblasts from *Col3a1* transgenic mice that did not show elevated levels of the unfolded protein response markers *Bip* and *Chop* [102]. In line with this evidence, a recent transcriptome analysis of dermal fibroblasts from *FKBP14*-kEDS patients did not reveal a high expression of genes associated with ER stress and unfolded protein response activation [67], though early data on this EDS cell type suggested an enlargement of ER [104]. Nevertheless, given that different reports highlighted the role of ER stress in the pathogenesis of several collagenopathies [27,108,109], further work is warranted to explore the possible ER perturbation as a disease mechanism of vEDS to identify novel potential therapeutic targets.

## 9. Hypermobile Ehlers‒Danlos Syndrome

Hypermobile EDS (hEDS, OMIM#130020), mainly characterized by generalized joint hypermobility and its complications, minor skin changes, and apparently segregating with an autosomal dominant pattern, is still without a known molecular basis. The phenotypic spectrum of hEDS is wide-ranging and heterogeneous and further complicated by multiple associated symptoms shared with other heritable or acquired (autoimmune) connective tissue disorders and chronic inflammatory systemic diseases [32,110,111].

Despite the significant advances in molecular genetic techniques, attempts to disclose the genetic cause(s) of hEDS have been so far inconclusive. Several studies struggled to define its genetic etiology but without compelling evidence, corroborating the hypothesis of a high genetic heterogeneity of the condition [112,113,114,115]. The introduction of more selective clinical criteria for hEDS in the novel classification aimed to minimize heterogeneity allows for the formation of homogeneous cohorts to facilitate scientific research to discover the underlying genetic cause(s) of the condition [32]. Nowadays, hEDS is considered at one end of a continuous spectrum of phenotypes, which originates from isolated non-syndromic joint hypermobility and passing through the recently defined hypermobility spectrum disorders (HSD) [111]. HSD refers to patients who present symptomatic joint hypermobility but do not fulfill the new diagnostic criteria of hEDS. Recently, given the clinical continuity between hEDS and HSD and our data on patients’ dermal fibroblasts [49], it was proposed that these disorders might be considered as a single entity, referred to as hEDS/HSD [116], as already occurred for the hypermobility type of EDS and joint hypermobility syndrome [117]. Until now, no validated biological biomarkers have been identified for recognizing hEDS/HSD, which are dominated by extremely variable phenotypes and chronic disability affecting patients’ quality of life [111,116].

In this intricate scenario, the integration of various biological knowledge could be an effective strategy to delineate molecular mechanisms contributing to the disease pathophysiology. Transcriptome and proteome profiling can be useful to reveal specific biological signatures, thus providing insights not only for the understanding of the pathomechanisms but also for the identification of reliable tools for therapeutic options [118,119,120,121,122].

Previous findings based on transcriptome and protein studies on a cohort of hEDS/HSD patient-derived dermal fibroblasts represent up to now the main effort to unravel their complex etiopathogenesis [48,123]. Proteome profiling of patients’ cells is currently ongoing to corroborate these data, since the gene expression profiling and cellular studies on patients’ fibroblasts provided significant clues that are likely relevant for the disease pathogenesis. In the following paragraphs we review our past findings and discuss some novel emerging aspects, offering future perspectives for molecular research in this field.

## 10. Pathological ECM Remodeling and Defective Cell-Cell Interactions in hEDS/HSD Cells

Although hEDS/HSD etiology remains elusive, patients’ skin fibroblasts show a disorganization of the ECM like that observed in cells derived from the other EDS types. In particular, hEDS/HSD, cEDS, and vEDS fibroblasts exhibit a marked disorganization of collagen and fibronectin ECM and their specific α2β1 and α5β1 integrin receptors and showed the preferential expression of the αvβ3 integrin [44,49,50,123].

Transcriptome of hEDS/HSD fibroblasts revealed the dysregulated expression of several genes encoding either ECM glycoproteins such as elastin (*ELN*) and sparc/osteonectin (*SPOCK*), ECM regulators, i.e., metalloproteinases (*MMP16*, *PAPPA2*) and transglutaminase (*TGM2*), or ECM associated secreted factors such as secreted frizzled-related protein 2 (*SFRP2*) and transforming growth factor alpha (*TGFA*). This ECM signature is in common with cEDS and vEDS cells, underlining that the matrix perturbation may act as a key driving factor for the EDS pathogenesis, irrespective of the underlying molecular defects [50].

Cells sense the intrinsic mechanical properties of the ECM and convert these stimuli into intracellular signals [124]. In addition to integrins that primarily mediate this cell response, intracellular signals may be triggered also through the cadherin superfamily, which are calcium-dependent transmembrane proteins forming complex adhesions and connect to the actin cytoskeleton via numerous proteins [125,126]. Interestingly, transcriptome profiling of hEDS/HSD cells revealed a differential expression of many adhesion molecule-encoding genes including members of cadherins and protocadherins, i.e., *CDH2*, *CDH10*, *PCDH9*, *PCDHB16*, *PCDHB18*, claudins (*CLDN11*), and desmosomes (desmoplakin, *DSP*), which are involved in the formation of specialized cell-cell junction complexes essential for maintaining epithelial integrity, morphogenesis, and tissue architecture [127,128]. Since these adhesion proteins can act as signaling modulators of intracellular pathways, such as Wnt, Hippo, NF-kB, JAK-STAT that are crucial for development and organogenesis [128], their altered expression could impact on multiple biological processes essential for embryogenesis and tissue homeostasis.

These transcriptional changes suggested a fibroblast-to-myofibroblast transition of hEDS/HSD cells. This phenomenon induces the formation of cells with muscle-like features that are characterized by increased cell contractility, formation of alpha smooth muscle actin (α-SMA)-stress fibers, together with the reorganization of cell-matrix and cell-cell contacts and cytoskeletal architecture [127,129,130,131]. Our in vitro studies confirmed the phenotypic conversion of hEDS/HSD fibroblasts into migrating myofibroblast-like cells, since they express the typical markers α-SMA and cadherin-11 and show augmented levels of the protease MMP9 and an altered expression of the inflammation mediators CYR61 and CTGF [49]. This phenotypic switch is elicited by a signal transduction pathway involving the αvβ3 integrin that signals through the integrin linked kinase (ILK) and the transcription factor Snail1 [49,50]. This myofibroblast-like phenotype observed in vitro might reflect a persistent in vivo inflammatory-like condition consistent with the patients’ systemic clinical manifestations, comprising gastrointestinal dysfunction, increased susceptibility to osteoarthritis, chronic generalized musculoskeletal pain, inflammatory soft-tissue lesions, and neurological features [110,111].

Activation of myofibroblasts is itself part of physiological wound repair following tissue injury, whereas in chronic injury and inflammatory fibrotic conditions their persistent activation exacerbates the disease progression [132,133,134,135]. During fibroblast-to-myofibroblast transition, a complex mechanochemical signaling is activated involving profibrotic secreted factors such as TGFβ and Wnt and ECM-degrading enzymes and intracellular effectors required for stress fiber contractility [127,131]. At molecular level, the cytokine TGFβ is considered the master regulator of profibrotic processes. A growing body of evidence has highlighted the regulation of the Wnt/β-catenin pathway by TGFβ as well as the involvement of their downstream molecular effectors in the fibroblast-to-myofibroblast transition and fibrotic responses [136].

As revealed by transcriptome analysis, several signaling pathways essential for cell growth and proliferation were found to be likely perturbed in hEDS/HSD fibroblasts, i.e., TGFβ, TNF, Jak-STAT, and PI3K-Akt [123]. Transcriptomics data also suggested the differential expression of different Wnt-related genes including the up-regulated frizzled receptor 3 (*FZD3*) and the down-regulated Wnt negative regulators *PRICKLE1* and *SFRP2*. *SFRP2*, the most down-regulated transcript in patients’ cells, acts as a critical Wnt modulator, since it directly binds to Wnt proteins and prevents their interactions with FZD receptors [137].

The synergistic crosstalk between TGFβ and Wnt signaling in the myofibroblast activation is documented as well as the inhibitory role of SFRP2 in the TGFβ-dependent myofibroblast formation and post-inflammatory fibrosis [138,139]. In this view, a possible involvement of these signaling pathways in the pathomechanisms of hEDS/HSD can be envisaged. Our findings may offer further clues to address important questions concerning the activation of these pathological mechanisms, and it remains to be clarified which growth factors, i.e., TGFβ, CTGF, and key regulatory pathways sustain the fibroblast-to-myofibroblast transition of hEDS/HSD cells.

## 11. Differential Expression of Genes Involved in Inflammatory, Immune, and Pain Response in hEDS/HSD Cells

Over the past few years, clinical research described the presence of comorbidities in hEDS/HSD patients, such as functional gastrointestinal and eosinophilic disorders [140,141], increased prevalence of asthma [142], and chronic pain syndromes, i.e., chronic fatigue, fibromyalgia, irritable bowel disease, and inflammatory joints conditions [111,143], though specific underlying causes and mechanisms remain to be explored. In this regard, transcriptome of hEDS/HSD cells revealed the aberrant transcription of a range of genes related to inflammation, pain, and immune responses, i.e., *AQP9*, *CFD*, *SPON2*, *PRLR*, and *NR4A* receptors, which might impair biological functions and molecular pathways with a potential role in the disease’s pathogenesis. Among them, patients’ cells showed the enhanced expression of *AQP9*, a member of the family of water-selective membrane channels that play a role both in antimicrobial defense and skin barrier permeability [144]. A high expression of this transporter was detected in synovial tissues and fibroblast-like synoviocytes from osteoarthritis and rheumatoid arthritis patients and may have a role in the pathogenesis of inflammatory synovitis [145,146].

In line with this finding, patients’ cells also showed increased mRNA levels of complement factor D (*CFD*), a component of the alternative complement pathway [147], which is involved in pathophysiological mechanisms of osteoarthritis and is considered as a potential predictive biomarker of joint pain in patients with hip and knee osteoarthritis [148,149,150].

In hEDS/HSD no reliable biomarkers have been identified. A previous study identified elevated basal serum tryptase levels due to increased *TPSAB1* copy number associated with hereditary alpha tryptasemia in individuals with multisystem complaints, i.e., joint hypermobility, sleep disruption, irritable bowel syndrome, body pain, headache, arthralgia, and chronic gastroesophageal reflux, partly overlapping with those frequently observed in hEDS/HSD patients [151]. In our hEDS/HSD patients, no elevated basal serum tryptase level was observed, suggesting the absence of the association between their clinical features and copy number variations in the *TPSAB1* gene.

Other inflammation-related genes dysregulated in hEDS/HSD include *SPON2*, up-regulated in patients’ cells, which encodes an ECM protein with multifunctional properties in the innate immune system and inflammatory cell recruitment [152,153,154], and *PRLR*, showing a decreased expression in hEDS/HSD cells, which encode the prolactin receptor implicated in inflammatory responses and immune cells regulation [155,156]. The prolactin-PRLR axis contributes to the activation of pain-related pathways through the sensitization of transient receptor potential channels that promote painful sensations [157,158]. Chronic pain represents a common complaint among hEDS/HSD patients affecting their quality of life [111,116], though specific molecular pathways or mediators of pain are still unknown.

As further evidence of unbalanced inflammatory responses in patients’ cells, transcriptome revealed a decreased expression of the *NR4A* nuclear receptors (*NR4A1*, *NR4A2*, *NR4A3*), which act as transcriptional regulators of inflammatory responses mediated by NF-kB signaling [159,160,161]. These receptors attenuate inflammatory events through inhibition of the NF-kB nuclear translocation and induction of the expression of its inhibitor NFKBIA, which, in turn, blocks the NF-kB nuclear localizing sequence [159]. The concomitant decreased mRNA levels of *NR4A1* and *NFKBIA* in patients’ cells may be related to aberrant NF-kB signaling.

Despite additional work being required to support these hypotheses, our findings depict the complex sequence of transcriptional events that should stimulate more investigations to provide new insights into the pathomechanisms underlying the molecular networks related to aberrant inflammatory responses associated with hEDS/HSD.

## 12. Emerging Aspects of hEDS/HSD Pathophysiology by microRNAs Profiling

Transcriptome analysis may be a valuable strategy also to delineate distinct molecular signatures related to the differential expression of microRNAs (miRNAs). miRNAs are small non-coding RNA molecules ranging from 20–25 nucleotides in length that act mainly as negative regulators of gene expression by promoting the degradation of target mRNAs or repressing their translation [162,163]. Aberrant miRNA expression has been reported in several pathological conditions including cancer, musculoskeletal disorders, painful peripheral neuropathies, and fibromyalgia [164,165,166].

Our previous expression profiling of hEDS/HSD fibroblasts identified 19 dysregulated miRNAs [123]. Here, we report some examples that might offer interesting clues about the possible involvement of miRNAs in the regulation of potential targets and molecular pathways related to inflammation and Wnt signaling that seem to have a role in the disease pathogenesis.

The most overexpressed miRNA in patients’ cells was the miR-378-3p, which is considered a modulator of the epithelial-to-mesenchymal transition and is associated with inflammation and fibrosis through the positive modulation of NF-kB and TNFα pathways [167,168]. miRNA-224, which is also up-regulated in hEDS/HSD cells, is associated with the activation of the Wnt/β-catenin signaling through the inhibition of the expression of glycogen synthase kinase 3β and SFRP2, which are known Wnt suppressors [169]. Therefore, it is likely reasonable to assume that the enhanced expression of this miRNA may contribute to the decreased mRNA level of *SFRP2* observed in hEDS/HSD cells, supporting the hypothesis that the aberrant signaling of the Wnt/β-catenin axis might play a role in the disease mechanisms of hEDS/HSD.

We previously demonstrated the involvement of the ILK in the fibroblast-to-myofibroblast switch of hEDS/HSD cells. The ILK acts downstream of the phosphatidylinositol 3-kinase signaling pathway and negatively regulates the action of the glycogen synthase kinase 3β by phosphorylation of a specific serine residue, further strengthening our assumption that Wnt/β-catenin signaling is involved in hEDS/HSD pathogenesis [49,50]. Consistently, patients’ fibroblasts show a reduced expression of the miRNA-23a, which is implicated in the Wnt pathway regulation as well, by inhibiting the expression of FDZ5 and FDZ7 receptors [170]. Decreased levels of this miRNA were also found in cerebrospinal fluid and serum of patients with fibromyalgia [171], a painful disorder in differential diagnosis with hEDS/HSD [172], which might suggest a possible involvement of specific miRNA signatures or a common disease pattern in both conditions. In addition, the altered expression of this miRNA in synovial fibroblasts of psoriatic arthritis patients, results in the enhanced expression of pro-inflammatory mediators and matrix degrading enzymes, further promoting joint degeneration and synovial inflammation [173].

Several studies highlighted the contribution of miRNAs in the modulation of the expression of ECM structural proteins and related signaling molecules, thus emphasizing the close relationship between ECM homeostasis and inflammatory pain related conditions [174,175,176].

As the expression of miRNAs can be modulated to mediate the expression of their target genes, in-depth in vitro studies on a large cohort of patients’ cells could provide further evidence on mechanisms of action of miRNAs and their impact on diverse target genes and altered pathways relevant for the pathophysiology of hEDS/HSD, thus offering new perspectives to identify potential molecular therapeutic targets.

## 13. Conclusions and Perspectives

**Figure 1:**
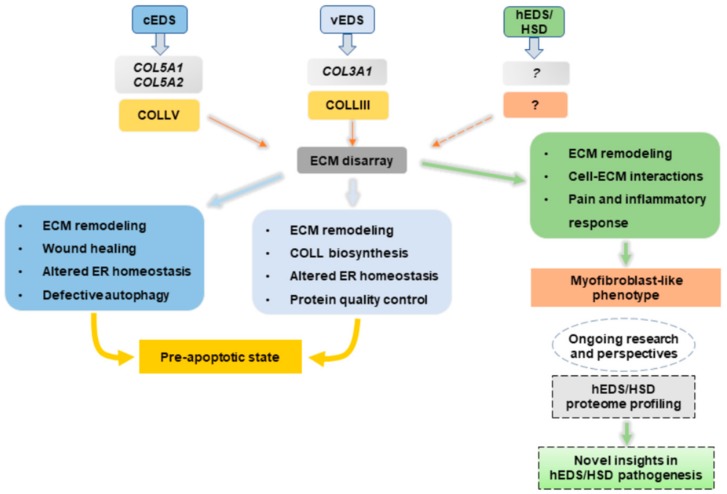
Schematic illustration summarizing the processes likely involved in the pathogenesis of cEDS, vEDS, and hEDS/HSD derived from transcriptome and in vitro studies of patients’ skin fibroblasts.

Transcriptome and in vitro analyses on cEDS, vEDS, and hEDS/HSD dermal fibroblasts expanded the knowledge about molecular mechanisms involved in the pathophysiology of these connective tissue disorders (Figure 1).

Our findings indicate that these cells share a deregulated expression of many matrix-related genes and a widespread disarray of several ECM structural constituents, thus highlighting the functional relevance of a proper organization and function of the ECM in providing stability to connective tissues. In cEDS and vEDS dermal fibroblasts, the pathological ECM turnover is directly caused by the underlying molecular defect causing abnormal expression of collagen V and collagen III, which, in turn, perturbs key physiological processes critical for collagen processing itself and to maintain cell homeostasis. In the absence of a known genetic etiology, the abnormal ECM organization present in hEDS/HSD cells may be a functional consequence of excessive remodeling due to increased levels of ECM-degrading enzymes and concomitant acquisition of a pro-inflammatory myofibroblast-like phenotype. hEDS/HSD transcriptome profiling for the first time has shed light on different pathobiological aspects of the disease. The dysregulated expression of genes involved in cell-matrix interactions and specific intracellular signaling pathways may have a role in the phenotypic switch of hEDS/HSD cells. Transcriptional changes of different genes and miRNAs involved in molecular pathways related to pain and inflammatory response might provide further clues to dissect the intricate biological events involved in chronic and musculoskeletal pain affecting hEDS/HSD patients. To deepen the knowledge on hEDS/HSD pathophysiology, proteome profiling of patients’ cells is currently ongoing to decipher the complex protein network and identify potential bioactive molecules involved in the disease pathogenesis to offer therapeutic options for hEDS/HSD patients.

## Figures and Tables

**Table 1:** EDS types grouped according to the underlying genetic defect and pathomechanisms.

EDS Type	IP	Gene	Protein
**Group A: disorders of collagen primary structure and collagen processing**			
Classical EDS (cEDS)	AD	Major: *COL5A1*, *COL5A2* Rare: *COL1A1*	COLLV COLLI
Vascular EDS (vEDS)	AD	*COL3A1*	COLLIII
Arthrochalasia EDS (aEDS)	AD	*COL1A1*, *COL1A2*	COLLI
Dermatosparaxis EDS (dEDS)	AR	*ADAMTS2*	ADAMTS-2
Cardiac-valvular EDS (cvEDS)	AR	*COL1A2*	COLLI
Classical-like 2 EDS ^A^ (cl2EDS)	AR	*AEBP1*	ACLP
**Group B: disorders of collagen folding and collagen cross-linking**			
Kyphoscoliotic EDS (kEDS)	AR	*PLOD1* *FKBP14*	LH1 FKBP22
**Group C: disorders of structure and function of myomatrix**			
Classical-like EDS (clEDS) Myopathic EDS (mEDS)	AR AD/AR	*TNXB* *COL12A1*	Tenascin X COLLXII
**Group D: disorders of glycosaminoglycan biosynthesis**			
Spondylodysplastic EDS (spEDS)	AR	*B4GALT7* *B3GALT6*	β4GalT7 β3GalT6
Musculocontractural EDS (mcEDS)	AR	*CHST14* *DSE*	D4ST1 DSE
**Group E: disorders of complement pathway**			
Periodontal EDS (pEDS)	AD	*C1R* *C1S*	C1r C1s
**Group F: disorders of intracellular processes**			
Spondylodysplastic EDS (spEDS)	AR	*SLC39A13*	ZIP13
Brittle Cornea Syndrome (BCS)	AR	*ZNF469* *PRDM5*	ZNF469 PRDM5
**EDS type molecularly unsolved**			
Hypermobile EDS (hEDS)	AD	Unknown	Unknown

^A^ New EDS variant recently defined in [33,34,35,36]. AD: autosomal dominant; AR: autosomal recessive; IP: inheritance pattern.

**Table 2:** Overview of the biological processes dysregulated in cEDS, vEDS, and hEDS patients’ dermal fibroblasts emerged from transcriptome and in vitro studies.

Insights in the Pathogenesis of cEDS, vEDS, and hEDS	Transcriptome Findings on Patients’ Fibroblasts			In Vitro Studies on Patients’ Fibroblasts		
Perturbed Biological Processes	cEDS	vEDS	hEDS	cEDS	vEDS	hEDS
ECM disorganization	+	+	+	+	+	+
Altered cell-matrix interactions	−	−	+	+	+	+
Disturbed cell-cell contacts	−	−	+	−	−	+
Fibroblast-to-myofibroblast transition	−	−	+	−	−	+
Altered inflammatory responses	+	−	+	−	na	+
Perturbed cell migration	+	+	−	+	+	+
Defective wound healing	+	+	−	+	na	na
Survival from anoikis	−	−	−	+	+	na
Collagens biosynthesis/processing	+	+	−	na	+	na
ER homeostasis/protein folding	+	+	−	na	+	na

+: detected by transcriptome or in vitro studies, −: not experimentally detected by transcriptome or in vitro studies, na: not ascertained.

## References

[1] Theocharis A.D., Manou D., Karamanos N.K. (2019). The extracellular matrix as a multitasking player in disease. FEBS J..

[2] Arseni L., Lombardi A., Orioli D. (2018). From Structure to Phenotype: Impact of Collagen Alterations on Human Health. Int. J. Mol. Sci..

[3] Murphy-Ullrich J.E., Sage E.H. (2014). Revisiting the matricellular concept. Matrix Biol..

[4] Hynes R.O., Naba A. (2012). Overview of the matrisome—An inventory of extracellular matrix constituents and functions. Cold Spring Harb. Perspect. Biol..

[5] Naba A., Clauser K.R., Ding H., Whittaker C.A., Carr S.A., Hynes R.O. (2016). The extracellular matrix: Tools and insights for the “omics” era. Matrix Biol..

[6] Myllyharju J., Kivirikko K.I. (2004). Collagens, modifying enzymes and their mutations in humans, flies and worms. Trends Genet..

[7] Nyström A., Bruckner-Tuderman L. (2019). Matrix molecules and skin biology. Semin. Cell Dev. Biol..

[8] Gjaltema R.A., Bank R.A. (2017). Molecular insights into prolyl and lysyl hydroxylation of fibrillar collagens in health and disease. Crit. Rev. Biochem. Mol. Biol..

[9] Sorushanova A., Delgado L.M., Wu Z., Shologu N., Kshirsagar A., Raghunath R., Mullen A.M., Bayon Y., Pandit A., Raghunath M. (2019). The Collagen Suprafamily: From Biosynthesis to Advanced Biomaterial Development. Adv. Mater..

[10] Malfait F. (2018). Vascular aspects of the Ehlers-Danlos Syndromes. Matrix Biol..

[11] Lenselink E.A. (2015). Role of fibronectin in normal wound healing. Int. Wound J..

[12] Iozzo R.V., Gubbiotti M.A. (2018). Extracellular matrix: The driving force of mammalian diseases. Matrix Biol..

[13] Karamanos N.K., Theocharis A.D., Neill T., Iozzo R.V. (2019). Matrix modeling and remodeling: A biological interplay regulating tissue homeostasis and diseases. Matrix Biol..

[14] Schwartz M.A. (2010). Integrins and extracellular matrix in mechanotransduction. Cold Spring Harb. Perspect. Biol..

[15] Campbell I.D., Humphries M.J. (2011). Integrin structure, activation, and interactions. Cold Spring Harb. Perspect. Biol..

[16] Frisch S.M., Francis H. (1994). Disruption of epithelial cell matrix interactions induces apoptosis. J. Cell Biol..

[17] Vlahakis A., Debnath J. (2017). The Interconnections between Autophagy and Integrin-Mediated Cell Adhesion. J. Mol. Biol..

[18] Lock R., Debnath J. (2008). Extracellular matrix regulation of autophagy. Curr. Opin. Cell Biol..

[19] Neill T., Schaefer L., Iozzo R.V. (2014). Instructive roles of extracellular matrix on autophagy. Am. J. Pathol..

[20] Settembre C., Cinque L., Bartolomeo R., Di Malta C., De Leonibus C., Forrester A. (2018). Defective collagen proteostasis and matrix formation in the pathogenesis of lysosomal storage disorders. Matrix Biol..

[21] Lamandé S.R., Bateman J.F. (2018). Collagen VI disorders: Insights on form and function in the extracellular matrix and beyond. Matrix Biol..

[22] Wilkinson S. (2019). Emerging Principles of Selective ER Autophagy. J. Mol. Biol..

[23] Bonnans C., Chou J., Werb Z. (2014). Remodelling the extracellular matrix in development and disease. Nat. Rev. Mol. Cell Biol..

[24] Frevert C.W., Felgenhauer J., Wygrecka M., Nastase M.V., Schaefer L. (2018). Danger-Associated Molecular Patterns Derived From the Extracellular Matrix Provide Temporal Control of Innate Immunity. J. Histochem. Cytochem..

[25] Genovese F., Karsdal M.A. (2016). Protein degradation fragments as diagnostic and prognostic biomarkers of connective tissue diseases: Understanding the extracellular matrix message and implication for current and future serological biomarkers. Expert Rev. Proteom..

[26] Bhattacharyya S., Midwood K.S., Yin H., Varga J. (2017). Toll-Like Receptor-4 Signaling Drives Persistent Fibroblast Activation and Prevents Fibrosis Resolution in Scleroderma. Adv. Wound Care.

[27] Lamandé S.R., Bateman J.F. (2019). Genetic Disorders of the Extracellular Matrix. Anat. Rec..

[28] Colombi M., Dordoni C., Chiarelli N., Ritelli M. (2015). Differential diagnosis and diagnostic flow chart of joint hypermobility syndrome/Ehlers-Danlos syndrome hypermobility type compared to other heritable connective tissue disorders. Am. J. Med. Genet. Part C.

[29] Bateman J.F., Boot-Handford R.P., Lamande S.R. (2009). Genetic diseases of connective tissues: Cellular and extra cellular effects of ECM mutations. Nat. Rev. Genet..

[30] Lu P., Takai K., Weaver V.M., Werb Z. (2011). Extracellular matrix degradation and remodeling in development and disease. Cold Spring Harb. Perspect. Biol..

[31] Karsdal M.A., Nielsen M.J., Sand J.M., Henriksen K., Genovese F., Bay-Jensen A.C., Smith V., Adamkewicz J.I., Christiansen C., Leeming D.J. (2013). Extracellular matrix remodeling: The common denominator in connective tissue diseases. Possibilities for evaluation and current understanding of the matrix as more than a passive architecture, but a key player in tissue failure. ASSAY Drug Dev. Technol..

[32] Malfait F., Francomano C., Byers P., Belmont J., Berglund B., Black J., Bloom L., Bowen J.M., Brady A.F., Burrows N.P. (2017). The 2017 international classification of the Ehlers-Danlos syndromes. Am. J. Med. Genet. C Semin. Med. Genet..

[33] Blackburn P.R., Xu Z., Tumelty K.E., Zhao R.W., Monis W.J., Harris K.G., Gass J.M., Cousin M.A., Boczek N.J., Mitkov M.V. (2018). Bi-allelic alterations in AEBP1 lead to defective collagen assembly and connective tissue structure resulting in a variant of Ehlers-Danlos syndrome. Am. J. Hum. Genet..

[34] Hebebrand M., Vasileiou G., Krumbiegel M., Kraus C., Uebe S., Ekici A.B., Thiel C.T., Reis A., Popp B. (2019). A biallelic truncating AEBP1 variant causes connective tissue disorder in two siblings. Am. J. Med. Genet. A.

[35] Syx D., De Wandele I., Symoens S., De Rycke R., Hougrand O., Voermans N., De Paepe A., Malfait F. (2019). Bi-allelic AEBP1 mutations in two patients with Ehlers-Danlos syndrome. Hum. Mol. Genet..

[36] Ritelli M., Cinquina V., Venturini M., Pezzaioli L., Formenti A.M., Chiarelli N., Colombi M. (2019). Expanding the Clinical and Mutational Spectrum of Recessive AEBP1-Related Classical-Like Ehlers-Danlos Syndrome. Genes..

[37] Maugeri A., De Paepe A., Malfait F., Jacobs J.W.G., Cornelissens L.J.M. (2018). Genetics and testing of Ehlers-Danlos syndrome and of differential diagnostic diseases. Ehlers-Danlos Syndrome: A Multidisciplinary Approach.

[38] Damme T.M., Syx D., Coucke P., Symoens S., De Paepe A., Malfait F. (2015). Genetics of the Ehlers–Danlos syndrome: More than collagen disorders. Expert Opin. Orphan Drugs.

[39] Bowen J.M., Sobey G.J., Burrows N.P., Colombi M., Lavallee M.E., Malfait F., Francomano C.A. (2017). Ehlers-Danlos syndrome, classical type. Am. J. Med. Genet. C Semin. Med. Genet..

[40] Symoens S., Syx D., Malfait F., Callewaert B., De Backer J., Vanakker O., Coucke P., De Paepe A. (2012). Comprehensive molecular analysis demonstrates type V collagen mutations in over 90% of patients with classic EDS and allows to refine diagnostic criteria. Hum. Mutat..

[41] Ritelli M., Dordoni C., Venturini M., Chiarelli N., Quinzani S., Traversa M., Zoppi N., Vascellaro A., Wischmeijer A., Manfredini E. (2013). Clinical and molecular characterization of 40 patients with classic Ehlers-Danlos syndrome: Identification of 18 COL5A1 and 2 COL5A2 novel mutations. Orphanet J. Rare Dis..

[42] Wenstrup R.J., Florer J.B., Brunskill E.W., Bell S.M., Chervoneva I., Birk D.E. (2004). Type V collagen controls the initiation of collagen fibril assembly. J. Biol. Chem..

[43] Mak K.M., Png C.Y., Lee D.J. (2016). Type V Collagen in Health, Disease, and Fibrosis. Anat. Rec..

[44] Zoppi N., Gardella R., De Paepe A., Barlati S., Colombi M. (2004). Human fibroblasts with mutations in COL5A1 and COL3A1 genes do not organize collagens and fibronectin in the extracellular matrix, down-regulate α2β1 integrin, and recruit αvβ3 instead of α5β1 integrin. J. Biol. Chem..

[45] Zoppi N., Barlati S., Colombi M. (2008). FAK-independent αvβ3 integrin-EGFR complexes rescue from anoikis matrix-defective fibroblasts. Biochim. Biophys. Acta.

[46] Zoppi N., Ritelli M., Colombi M. (2012). Type III and V collagens modulate the expression and assembly of EDA+fibronectin in the extracellular matrix of defective Ehlers-Danlos syndrome fibroblasts. Biochim. Biophys. Acta.

[47] Chiarelli N., Carini G., Zoppi N., Ritelli M., Colombi M. (2019). Molecular insights in the pathogenesis of classical Ehlers-Danlos syndrome from transcriptome-wide expression profiling of patients’ skin fibroblasts. PLoS ONE.

[48] Viglio S., Zoppi N., Sangalli A., Gallanti A., Barlati S., Mottes M., Colombi M., Valli M. (2008). Rescue of migratory defects of Ehlers-Danlos syndrome fibroblasts in vitro by type V collagen but not insulin-like binding protein-1. J. Investig. Dermatol..

[49] Zoppi N., Chiarelli N., Binetti S., Ritelli M., Colombi M. (2018). Dermal fibroblast-to-myofibroblast transition sustained by αvβ3 integrin-ILK-Snail1/Slug signaling is a common feature for hypermobile Ehlers-Danlos syndrome and hypermobility spectrum disorders. Biochim. Biophys. Acta.

[50] Zoppi N., Chiarelli N., Ritelli M., Colombi M. (2018). Multifaced Roles of the αvβ3 Integrin in Ehlers-Danlos and Arterial Tortuosity Syndromes’ Dermal Fibroblasts. Int. J. Mol. Sci..

[51] DeNigris J., Yao Q., Birk E.K., Birk D.E. (2016). Altered dermal fibroblast behavior in a collagen V haploinsufficient murine model of classic Ehlers-Danlos syndrome. Connect. Tissue Res..

[52] Park A.C., Phan N., Massoudi D., Liu Z., Kernien J.F., Adams S.M., Davidson J.M., Birk D.E., Liu B., Greenspan D.S. (2017). Deficits in Col5a2 Expression Result in Novel Skin and Adipose Abnormalities and Predisposition to Aortic Aneurysms and Dissections. Am. J. Pathol..

[53] Rousselle P., Montmasson M., Garnier C. (2019). Extracellular matrix contribution to skin wound re-epithelialization. Matrix Biol..

[54] Tracy L.E., Minasian R.A., Caterson E.J. (2016). Extracellular Matrix and Dermal Fibroblast Function in the Healing Wound. Adv. Wound Care.

[55] Chester D., Brown A.C. (2017). The role of biophysical properties of provisional matrix proteins in wound repair. Matrix Biol..

[56] Wang W., Li P., Li W., Jiang J., Cui Y., Li S., Wang Z. (2017). Osteopontin activates mesenchymal stem cells to repair skin wound. PLoS ONE.

[57] Icer M.A., Gezmen-Karadag M. (2018). The multiple functions and mechanisms of osteopontin. Clin. Biochem..

[58] Ross F.P., Chappel J., Alvarez J., Sander D., Butler W., Farach-Carson M., Mintz K., Robey P.G., Teitelbaum S., Cheresh D. (1993). Interactions between the bone matrix proteins osteopontin and bone sialoprotein and the osteoclast integrin alpha v beta 3 potentiate bone resorption. J. Biol. Chem..

[59] Giachelli C.M., Steitz S. (2000). Osteopontin: A versatile regulator of inflammation and biomineralization. Matrix Biol..

[60] Liaw L., Birk D.E., Ballas C.B., Whitsitt J.S., Davidson J.M., Hogan B.L. (1998). Altered wound healing in mice lacking a functional osteopontin gene (spp1). J. Clin. Investig..

[61] Maruhashi T. (2010). Interaction between periostin and BMP-1 promotes proteolytic activation of lysyl oxidase. J. Biol. Chem..

[62] Egbert M., Ruetze M., Sattler M., Wenck H., Gallinat S., Lucius R., Weise J.M. (2014). The matricellular protein periostin contributes to proper collagen function and is downregulated during skin aging. J. Dermatol. Sci..

[63] González-González L., Alonso J. (2018). Periostin: A Matricellular Protein with Multiple Functions in Cancer Development and Progression. Front. Oncol..

[64] Li G., Jin R., Norris R.A., Zhang L., Yu S., Wu F., Markwald R.R., Nanda A., Conway S.J., Smyth S.S. (2010). Periostin mediates vascular smooth muscle cell migration through the integrins alphavbeta3 and alphavbeta5 and focal adhesion kinase (FAK) pathway. Atherosclerosis.

[65] Walker J.T., McLeod K., Kim S., Conway S.J., Hamilton D.W. (2016). Periostin as a multifunctional modulator of the wound healing response. Cell Tissue Res..

[66] Nunomura S., Nanri Y., Ogawa M., Arima K., Mitamura Y., Yoshihara T., Hasuwa H., Conway S.J., Izuhara K. (2018). Constitutive overexpression of periostin delays wound healing in mouse skin. Wound Repair Regen..

[67] Lim P.J., Lindert U., Opitz L., Hausser I., Rohrbach M., Giunta C. (2019). Transcriptome Profiling of Primary Skin Fibroblasts Reveal Distinct Molecular Features Between PLOD1-and FKBP14-Kyphoscoliotic Ehlers-Danlos Syndrome. Genes.

[68] Penta K., Varner J.A., Liaw L., Hidai C., Schatzman R., Quertermous T. (1999). Del1 induces integrin signaling and angiogenesis by ligation of alphaVbeta3. J. Biol. Chem..

[69] Shen W., Zhu S., Qin H., Zhong M., Wu J., Zhang R., Song H. (2017). EDIL3 knockdown inhibits retinal angiogenesis through the induction of cell cycle arrest in vitro. Mol. Med. Rep..

[70] Wang Z., Boyko T., Tran M.C., LaRussa M., Bhatia N., Rashidi V., Longaker M.T., Yang G.P. (2018). DEL1 protects against chondrocyte apoptosis through integrin binding. J. Surg. Res..

[71] Hajishengallis G., Chavakis T. (2019). DEL-1-Regulated Immune Plasticity and Inflammatory Disorders. Trends Mol. Med..

[72] Brandt K., Grunler J., Brismar K., Wang J. (2015). Effects of IGFBP-1 and IGFBP-2 and their fragments on migration and IGF-induced proliferation of human dermal fibroblasts. Growth Horm. IGF Res..

[73] Katayama H., Tamai K., Shibuya R., Nakamura M., Mochizuki M., Yamaguchi K., Kawamura S., Tochigi T., Sato I., Okanishi T. (2017). Long non-coding RNA HOTAIR promotes cell migration by upregulating insulin growth factor-binding protein 2 in renal cell carcinoma. Sci. Rep..

[74] Ahmad S., Bhatia K., Kindelin A., Ducruet A.F. (2019). The Role of Complement C3a Receptor in Stroke. Neuromol. Med..

[75] Korkmaz H.I., Krijnen P.A.J., Ulrich M.M.W., de Jong E., van Zuijlen P.P.M., Niessen H.W.M. (2017). The role of complement in the acute phase response after burns. Burns.

[76] Rafail S., Kourtzelis I., Foukas P.G., Markiewski M.M., DeAngelis R.A., Guariento M., Ricklin D., Grice E.A., Lambris J.D. (2015). Complement deficiency promotes cutaneous wound healing in mice. J. Immunol..

[77] Colombi M., Dordoni C., Venturini M., Ciaccio C., Morlino S., Chiarelli N., Zanca A., Calzavara-Pinton P., Zoppi N., Castori M. (2017). Spectrum of mucocutaneous, ocular and facial features and delineation of novel presentations in 62 classical Ehlers-Danlos syndrome patients. Clin. Genet..

[78] Smith M., Wilkinson S. (2017). ER homeostasis and autophagy. Essays Biochem..

[79] Wong M.Y., Shoulders M.D. (2019). Targeting defective proteostasis in the collagenopathies. Curr. Opin. Chem. Biol..

[80] Moon H.W., Han H.G., Jeon Y.J. (2018). Protein Quality Control in the Endoplasmic Reticulum and Cancer. Int. J. Mol. Sci..

[81] Mogk A., Bukau B., Kampinga H.H. (2018). Cellular Handling of Protein Aggregates by Disaggregation Machines. Mol. Cell.

[82] Wilkinson S. (2019). ER-phagy: Shaping up and destressing the endoplasmic reticulum. FEBS J..

[83] Smith M.D., Harley M.E., Kemp A.J., Wills J., Lee M., Arends M., von Kriegsheim A., Behrends C., Wilkinson S. (2018). CCPG1 is a non-canonical autophagy cargo receptor essential for ER-phagy and pancreatic ER proteostasis. Dev. Cell.

[84] Forrester A., De Leonibus C., Grumati P., Fasana E., Piemontese M., Staiano L., Fregno I., Raimondi A., Marazza A., Bruno G. (2019). A selective ER-phagy exerts procollagen quality control via a Calnexin-FAM134B complex. EMBO J..

[85] Ishida Y., Yamamoto A., Kitamura A., Lamandé S.R., Yoshimori T., Bateman J.F., Kubota H., Nagata K. (2009). Autophagic elimination of misfolded procollagen aggregates in the endoplasmic reticulum as a means of cell protection. Mol. Biol. Cell.

[86] Nagahama M., Suzuki M., Hamada Y., Hatsuzawa K., Tani K., Yamamoto A., Tagaya M. (2003). SVIP is a novel VCP/p97-interacting protein whose expression causes cell vacuolation. Mol. Biol. Cell.

[87] Ballar P., Zhong Y., Nagahama M., Tagaya M., Shen Y., Fang S. (2007). Identification of SVIP as an endogenous inhibitor of endoplasmic reticulum-associated degradation. J. Biol. Chem..

[88] Wang Y., Ballar P., Zhong Y., Zhang X., Liu C., Zhang Y.J., Monteiro M.J., Li J., Fang S. (2011). SVIP induces localization of p97/VCP to the plasma and lysosomal membranes and regulates autophagy. PLoS ONE.

[89] Jia D., Wang Y.Y., Wang P., Huang Y., Liang D.Y., Wang D., Cheng C., Zhang C., Guo L., Liang P. (2019). SVIP alleviates CCl4-induced liver fibrosis via activating autophagy and protecting hepatocytes. Cell Death Dis..

[90] Fung C., Lock R., Gao S., Salas E., Debnath J. (2008). Induction of autophagy during extracellular matrix detachment promotes cell survival. Mol. Biol. Cell.

[91] Kawano S., Torisu T., Esaki M., Torisu K., Matsuno Y., Kitazono T. (2017). Autophagy promotes degradation of internalized collagen and regulates distribution of focal adhesions to suppress cell adhesion. Biol. Open.

[92] Byers P.H., Belmont J., Black J., De Backer J., Frank M., Jeunemaitre X., Johnson D., Pepin M., Robert L., Sanders L. (2017). Diagnosis, natural history, and management in vascular Ehlers-Danlos syndrome. Am. J. Med. Genet. C Semin. Med. Genet..

[93] Ritelli M., Rovati C., Venturini M., Chiarelli N., Cinquina V., Castori M., Colombi M. Application of the 2017 criteria for vascular Ehlers-Danlos syndrome in 50 patients ascertained according to the Villefranche nosology. Clin. Genet.

[94] Pepin M., Schwarze U., Superti-Furga A., Byers P.H. (2000). Clinical and genetic features of Ehlers-Danlos syndrome type IV, the vascular type. N. Engl. J. Med..

[95] Smith L.T., Schwarze U., Goldstein J., Byers P.H. (1997). Mutations in the COL3A1 gene result in the Ehlers-Danlos syndrome type IV and alterations in the size and distribution of the major collagen fibrils of the dermis. J. Investig. Dermatol..

[96] Liu X., Wu H., Byrne M., Krane S., Jaenisch R. (1997). Type III collagen is crucial for collagen I fibrillogenesis and for normal cardiovascular development. Proc. Natl. Acad. Sci. USA.

[97] Chiarelli N., Carini G., Zoppi N., Ritelli M., Colombi M. (2018). Transcriptome analysis of skin fibroblasts with dominant negative COL3A1 mutations provides molecular insights into the etiopathology of vascular Ehlers-Danlos syndrome. PLoS ONE.

[98] Sengle G., Sakai L.Y. (2015). The fibrillin microfibril scaffold: A niche for growth factors and mechanosensation?. Matrix Biol..

[99] Schiavinato A., Keene D.R., Wohl A.P., Corallo D., Colombatti A., Wagener R., Paulsson M., Bonaldo P., Sengle G. (2016). Targeting of EMILIN-1 and EMILIN-2 to fibrillin microfibrils facilitates their incorporation into the extracellular matrix. J. Investig. Dermatol..

[100] Boregowda R., Paul E., White J., Ritty T.M. (2008). Bone and soft connective tissue alterations result from loss of fibrillin-2 expression. Matrix Biol..

[101] Brinckmann J., Hunzelmann N., Kahle B., Rohwedel J., Kramer J., Gibson M.A., Hubmacher D., Reinhardt D.P. (2010). Enhanced fibrillin-2 expression is a general feature of wound healing and sclerosis: Potential alteration of cell attachment and storage of TGF-beta. Lab. Investig..

[102] D’Hondt S., Guillemyn B., Syx D., Symoens S., De Rycke R., Vanhoutte L., Toussaint W., Lambrecht B.N., De Paepe A., Keene D.R. (2018). Type III collagen affects dermal and vascular collagen fibrillogenesis and tissue integrity in a mutant Col3a1 transgenic mouse model. Matrix Biol..

[103] Ishikawa Y., Bächinger H.P. (2014). A substrate preference for the rough endoplasmic reticulum resident protein FKBP22 during collagen biosynthesis. J. Biol. Chem..

[104] Baumann M., Giunta C., Krabichler B., Rüschendorf F., Zoppi N., Colombi M., Bittner R.E., Quijano-Roy S., Muntoni F., Cirak S. (2012). Mutations in FKBP14 cause a variant of Ehlers-Danlos syndrome with progressive kyphoscoliosis, myopathy, and hearing loss. Am. J. Hum. Genet..

[105] Soares Moretti A., Martins Laurindo F.R. (2017). Protein disulfide isomerases: Redox connections in and out of the endoplasmic reticulum. Arch. Biochem. Biophys..

[106] Szegezdi E., Macdonald D.C., Ni Chonghaile T., Gupta S., Samali A. (2009). Bcl-2 family on guard at the ER. Am. J. Physiol.-Cell Physiol..

[107] Sisinni L., Pietrafesa M., Lepore S., Maddalena F., Condelli V., Esposito F., Landriscina M. (2019). Endoplasmic Reticulum Stress and Unfolded Protein Response in Breast Cancer: The Balance between Apoptosis and Autophagy and Its Role in Drug Resistance. Int. J. Mol. Sci..

[108] Boot-Handford R.P., Briggs M.D. (2010). The unfolded protein response and its relevance to connective tissue diseases. Cell Tissue Res..

[109] Hughes A., Oxford A.E., Tawara K., Jorcyk C.L., Oxford J.T. (2017). Endoplasmic Reticulum Stress and Unfolded Protein Response in Cartilage Pathophysiology; Contributing Factors to Apoptosis and Osteoarthritis. Int. J. Mol. Sci..

[110] Tinkle B., Castori M., Berglund B., Cohen H., Grahame R., Kazkaz H., Levy H. (2017). Hypermobile Ehlers-Danlos syndrome (a.k.a. Ehlers-Danlos syndrome type III and Ehlers-Danlos syndrome hypermobility type): Clinical description and natural history. Am. J. Med. Genet. C Semin. Med. Genet..

[111] Castori M., Tinkle B., Levy H., Grahame R., Malfait F., Hakim A. (2017). A framework for the classification of joint hypermobility and related conditions. Am. J. Med. Genet. C Semin. Med. Genet..

[112] Schalkwijk J., Zweers M.C., Steijlen P.M., Dean W.B., Taylor G., van Vlijmen I.M., van Haren B., Miller W.L., Bristow J. (2001). A recessive form of the Ehlers-Danlos syndrome caused by tenascin-X deficiency. N. Engl. J. Med..

[113] Merke D.P., Chen W., Morissette R., Xu Z., Van Ryzin C., Sachdev V., Hannoush H., Shanbhag S.M., Acevedo A.T., Nishitani M. (2013). Tenascin-X haploinsufficiency associated with Ehlers-Danlos syndrome in patients with congenital adrenal hyperplasia. J. Clin. Endocrinol. Metab..

[114] Morissette R., Chen W., Perritt A.F., Dreiling J.L., Arai A.E., Sachdev V., Hannoush H., Mallappa A., Xu Z., McDonnell N.B. (2015). Broadening the Spectrum of Ehlers Danlos Syndrome in Patients with Congenital Adrenal Hyperplasia. J. Clin. Endocrinol. Metab..

[115] Syx D., Symoens S., Steyaert W., De Paepe A., Coucke P.J., Malfait F. (2015). Ehlers-Danlos Syndrome, Hypermobility Type, Is Linked to Chromosome 8p22-8p21.1 in an Extended Belgian Family. Dis. Markers.

[116] Copetti M., Morlino S., Colombi M., Grammatico P., Fontana A., Castori M. (2019). Severity classes in adults with hypermobile Ehlers-Danlos syndrome/hypermobility spectrum disorders: A pilot study of 105 Italian patients. Rheumatology.

[117] Castori M., Dordoni C., Valiante M., Sperduti I., Ritelli M., Morlino S., Chiarelli N., Celletti C., Venturini M., Camerota F. (2014). Nosology and inheritance pattern(s) of joint hypermobility syndrome and Ehlers-Danlos syndrome, hypermobility type: A study of intrafamilial and interfamilial variability in 23 Italian pedigrees. Am. J. Med. Genet. A.

[118] Ritelli M., Chiarelli N., Zoppi N., Dordoni C., Quinzani S., Traversa M., Venturini M., Calzavara-Pinton P., Colombi M. (2014). Insights in the etiopathology of galactosyltransferase II (GalT-II) deficiency from transcriptome-wide expression profiling of skin fibroblasts of two sisters with compound heterozygosity for two novel B3GALT6 mutations. Mol. Genet. Metab. Rep..

[119] Zoppi N., Chiarelli N., Cinquina V., Ritelli M., Colombi M. (2015). GLUT10 deficiency leads to oxidative stress and non-canonical αvβ3 integrin-mediated TGFβ signaling associated with extracellular matrix disarray in arterial tortuosity syndrome skin fibroblasts. Hum. Mol. Genet..

[120] Manzoni C., Kia D.A., Vandrovcova J., Hardy J., Wood N.W., Lewis P.A., Ferrari R. (2018). Genome, transcriptome and proteome: The rise of omics data and their integration in biomedical sciences. Brief. Bioinform..

[121] Casamassimi A., Federico A., Rienzo M., Esposito S., Ciccodicola A. (2017). Transcriptome Profiling in Human Diseases: New Advances and Perspectives. Int. J. Mol. Sci..

[122] Ideozu J.E., Zhang X., McColley S., Levy H. (2019). Transcriptome Profiling and Molecular Therapeutic Advances in Cystic Fibrosis: Recent Insights. Genes.

[123] Chiarelli N., Carini G., Zoppi N., Dordoni C., Ritelli M., Venturini M., Castori M., Colombi M. (2016). Transcriptome-wide expression profiling in skin fibroblasts of patients with joint hypermobility syndrome/Ehlers-Danlos syndrome hypermobility type. PLoS ONE.

[124] Jansen K.A., Atherton P., Ballestrem C. (2017). Mechanotransduction at the cell-matrix interface. Semin. Cell Dev. Biol..

[125] Freedman B.R., Bade N.D., Riggin C.N., Zhang S., Haines P.G., Ong K.L., Janmey P.A. (2015). The (dys) functional extracellular matrix. Biochim. Biophys. Acta.

[126] Kalluri R., Neilson E.R. (2003). Epithelial-mesenchymal transition and its implications for fibrosis. J. Clin. Investig..

[127] Lamouille S., Xu J., Derynck R. (2014). Molecular mechanisms of epithelial-mesenchymal transition. Nat. Rev. Mol. Cell Biol..

[128] McCrea P.D., Maher M.T., Gottardi C.J. (2015). Nuclear signaling from cadherin adhesion complexes. Curr. Top. Dev. Biol..

[129] Hinz B., Gabbiani G. (2003). Cell-matrix and cell-cell contacts of myofibroblasts: Role in connective tissue remodeling. Thromb. Haemost..

[130] Michalik M., Wójcik-Pszczoła K., Paw M., Wnuk D., Koczurkiewicz P., Sanak M., Pękala E., Madeja Z. (2018). Fibroblast-to-myofibroblast transition in bronchial asthma. Cell. Mol. Life Sci..

[131] Hinz B., McCulloch C.A., Coelho N.M. (2019). Mechanical regulation of myofibroblast phenoconversion and collagen contraction. Exp. Cell Res..

[132] Pakshir P., Hinz B. (2018). The big five in fibrosis: Macrophages, myofibroblasts, matrix, mechanics, and miscommunication. Matrix Biol..

[133] Mullenbrock S., Liu F., Szak S., Hronowski X., Gao B., Juhasz P., Sun C., Liu M., McLaughlin H., Xiao Q. (2018). Systems Analysis of Transcriptomic and Proteomic Profiles Identifies Novel Regulation of Fibrotic Programs by miRNAs in Pulmonary Fibrosis Fibroblasts. Genes.

[134] Van Caam A., Vonk M., van den Hoogen F., van Lent P., van der Kraan P. (2018). Unraveling SSc Pathophysiology; The Myofibroblast. Front. Immunol..

[135] Watanabe T., Baker Frost D.A., Mlakar L., Heywood J., da Silveira W.A., Hardiman G., Feghali-Bostwick C. (2019). A Human Skin Model Recapitulates Systemic Sclerosis Dermal Fibrosis and Identifies COL22A1 as a TGFβ Early Response Gene that Mediates Fibroblast to Myofibroblast Transition. Genes.

[136] Działo E., Tkacz K., Błyszczuk P. (2018). Crosstalk between the TGF-β and WNT signalling pathways during cardiac fibrogenesis. Acta Biochim. Pol..

[137] Cruciat C.M., Niehrs C. (2013). Secreted and transmembrane wnt inhibitors and activators. Cold Spring Harb. Perspect. Biol..

[138] Carthy J.M., Garmaroudi F.S., Luo Z., McManus B.M. (2011). Wnt3a induces myofibroblast differentiation by upregulating TGF-β signaling through SMAD2 in a β-catenin-dependent manner. PLoS ONE.

[139] Blyszczuk P., Müller-Edenborn B., Valenta T., Osto E., Stellato M., Behnke S., Glatz K., Basler K., Lüscher T.F., Distler O. (2017). Transforming growth factor-β-dependent Wnt secretion controls myofibroblast formation and myocardial fibrosis progression in experimental autoimmune myocarditis. Eur. Heart J..

[140] Abonia J.P., Wen T., Stucke E.M., Grotjan T., Griffith M.S., Kemme K.A., Collins M.H., Putnam P.E., Franciosi J.P., Von Tiehl K.F. (2013). High prevalence of eosinophilic esophagitis in patients with inherited connective tissue disorders. J. Allergy Clin. Immunol..

[141] Fikree A., Grahame R., Aktar R., Farmer A.D., Hakim A.J., Morris J.K., Knowles C.H., Aziz Q. (2013). A prospective evaluation of undiagnosed joint hypermobility syndrome in patients with gastrointestinal symptoms. Clin. Gastroenterol. Hepatol..

[142] Morgan A.W., Pearson S.B., Davies S., Gooi H.C., Bird H.A. (2007). Asthma and airway collapse in two heritable disorders of connective tissue. Ann. Rheum. Dis..

[143] Rodgers K.R., Gui J., Dinulos M.B., Chou R.C. (2017). Ehlers-Danlos syndrome hypermobility type is associated with rheumatic diseases. Sci. Rep..

[144] Grether-Beck S., Felsner I., Brenden H., Kohne Z., Majora M., Marini A., Jaenicke T., Rodriguez-Martin M., Trullas C., Hupe M. (2012). Urea uptake enhances barrier function and antimicrobial defense in humans by regulating epidermal gene expression. J. Investig. Dermatol..

[145] Nagahara M., Waguri-Nagaya Y., Yamagami T., Aoyama M., Tada T., Inoue K., Asai K., Otsuka T. (2010). TNF-alpha-induced aquaporin 9 in synoviocytes from patients with OA and RA. Rheumatology.

[146] Takeuchi K., Hayashi S., Matumoto T., Hashimoto S., Takayama K., Chinzei N., Kihara S., Haneda M., Kirizuki S., Kuroda Y. (2018). Downregulation of aquaporin 9 decreases catabolic factor expression through nuclear factor-κB signaling in chondrocytes. Int. J. Mol. Med..

[147] Xu Y., Ma M., Ippolito G.C., Schroeder H.W., Carroll M.C., Volanakis J.E. (2001). Complement activation in factor D-deficient mice. Proc. Natl. Acad. Sci. USA.

[148] Kluzek S., Arden N.K., Newton J. (2015). Adipokines as potential prognostic biomarkers in patients with acute knee injury. Biomarkers.

[149] Martel-Pelletier J., Raynauld J.P., Dorais M., Abram F., Pelletier J.P. (2016). The levels of the adipokines adipsin and leptin are associated with knee osteoarthritis progression as assessed by MRI and incidence of total knee replacement in symptomatic osteoarthritis patients: A post hoc analysis. Rheumatology.

[150] Chandran V., Abji F., Perruccio A.V., Gandhi R., Li S., Cook R.J., Gladman D.D. (2019). Serum-based soluble markers differentiate psoriatic arthritis from osteoarthritis. Ann. Rheum. Dis..

[151] Lyons J.J., Yu X., Hughes J.D., Le Q.T., Jamil A., Bai Y., Ho N., Zhao M., Liu Y., O’Connell M.P. (2016). Elevated basal serum tryptase identifies a multisystem disorder associated with increased TPSAB1 copy number. Nat. Genet..

[152] He Y.W., Li H., Zhang J., Hsu C.L., Lin E., Zhang N., Guo J., Forbush K.A., Bevan M.J. (2004). The extracellular matrix protein mindin is a pattern-recognition molecule for microbial pathogens. Nat. Immunol..

[153] Jia W., Li H., He Y.W. (2005). The extracellular matrix protein mindin serves as an integrin ligand and is critical for inflammatory cell recruitment. Blood.

[154] Liu Y.S., Wang L.F., Cheng X.S., Huo Y.N., Ouyang X.M., Liang L.Y., Lin Y., Wu J.F., Ren J.L., Guleng B. (2019). The pattern-recognition molecule mindin binds integrin Mac-1 to promote macrophage phagocytosis via Syk activation and NF-κB p65 translocation. J. Cell. Mol. Med..

[155] Clapp C., Adán N., Ledesma-Colunga M.G., Solís-Gutiérrez M., Triebel J., Martínez de la Escalera G. (2016). The role of the prolactin/vasoinhibin axis in rheumatoid arthritis: An integrative overview. Cell. Mol. Life Sci..

[156] Ledesma-Colunga M.G., Adán N., Ortiz G., Solís-Gutiérrez M., López-Barrera F., Martínez de la Escalera G., Clapp C. (2017). Prolactin blocks the expression of receptor activator of nuclear factor κB ligand and reduces osteoclastogenesis and bone loss in murine inflammatory arthritis. Arthritis Res. Ther..

[157] Patil M.J., Ruparel S.B., Henry M.A., Akopian A.N. (2013). Prolactin regulates TRPV1, TRPA1, and TRPM8 in sensory neurons in a sex-dependent manner: Contribution of prolactin receptor to inflammatory pain. Am. J. Physiol. Endocrinol. Metab..

[158] Basso L., Altier C. (2017). Transient Receptor Potential Channels in neuropathic pain. Curr. Opin. Pharmacol..

[159] Rodríguez-Calvo R., Tajes M., Vázquez-Carrera M. (2017). The NR4A subfamily of nuclear receptors: Potential new therapeutic targets for the treatment of inflammatory diseases. Expert Opin. Ther. Targets.

[160] Murphy E.P., Crean D. (2015). Molecular Interactions between NR4A Orphan Nuclear Receptors and NF-Kb Are Required for Appropriate Inflammatory Responses and Immune Cell Homeostasis. Biomolecules.

[161] Banno A., Lakshmi S.P., Reddy A.T., Kim S.C., Reddy R.C. (2019). Key Functions and Therapeutic Prospects of Nur77 in Inflammation Related Lung Diseases. Am. J. Pathol..

[162] Fernandes J.C.R., Acuña S.M., Aoki J.I., Floeter-Winter L.M., Muxel S.M. (2019). Long Non-Coding RNAs in the Regulation of Gene Expression: Physiology and Disease. Noncoding RNA.

[163] Filipowicz W., Bhattacharyya S.N., Sonenberg N. (2008). Mechanisms of post-transcriptional regulation by microRNAs: Are the answers insight?. Nat. Rev. Genet.

[164] Leinders M., Üçeyler N., Thomann A., Sommer C. (2017). Aberrant microRNA expression in patients with painful peripheral neuropathies. J. Neurol. Sci..

[165] Greco S., Cardinali B., Falcone G., Martelli F. (2018). Circular RNAs in Muscle Function and Disease. Int. J. Mol. Sci..

[166] D’Agnelli S., Arendt-Nielsen L., Gerra M.C., Zatorri K., Boggiani L., Baciarello M., Bignami E. (2019). Fibromyalgia: Genetics and epigenetics insights may provide the basis for the development of diagnostic biomarkers. Mol. Pain.

[167] Kim J., Hyun J., Wang S., Lee C., Jung Y. (2018). MicroRNA-378 is involved in hedgehog-driven epithelial-to-mesenchymal transition in hepatocytes of regenerating liver. Cell Death Dis..

[168] Zhang T., Hu J., Wang X., Zhao X., Li Z., Niu J., Steer C.J., Zheng G., Song G. (2019). MicroRNA-378 promotes hepatic inflammation and fibrosis via modulation of the NF-κB-TNFα pathway. J. Hepatol..

[169] Xiao Z., Deng D., He L., Jiao H., Ye Y., Liang L., Ding Y., Liao W. (2016). MicroRNA-224 sustains Wnt/β-catenin signaling and promotes aggressive phenotype of colorectal cancer. J. Exp. Clin. Cancer Res..

[170] Peng Y., Zhang X., Feng X., Fan X., Jin Z. (2017). The crosstalk between microRNAs and the Wnt/β-catenin signaling pathway in cancer. Oncotarget.

[171] Bjersing J.L., Lundborg C., Bokarewa M.I., Mannerkorpi K. (2013). Profile of cerebrospinal microRNAs in fibromyalgia. PLoS ONE.

[172] Chopra P., Tinkle B., Hamonet C., Brock I., Gompel A., Bulbena A., Francomano C. (2017). Pain management in the Ehlers-Danlos syndromes. Am. J. Med. Genet. C Semin. Med. Genet..

[173] Wade S.M., Trenkmann M., McGarry T., Canavan M., Marzaioli V., Wade S.C., Veale D.J., Fearon U. (2019). Altered expression of microRNA-23a in psoriatic arthritis modulates synovial fibroblast pro-inflammatory mechanisms via phosphodiesterase 4B. J. Autoimmun..

[174] Tajerian M., Clark J.D. (2015). The role of the extracellular matrix in chronic pain following injury. Pain.

[175] Parisien M., Samoshkin A., Tansley S.N., Piltonen M.H., Martin L.J., El-Hachem N., Dagostino C., Allegri M., Mogil J.S., Khoutorsky A. (2019). Genetic pathway analysis reveals a major role for extracellular matrix organization in inflammatory and neuropathic pain. Pain.

[176] Andersen H.H., Duroux M., Gazerani P. (2014). MicroRNA as modulators and biomarkers of inflammatory and neuropathic pain conditions. Neurobiol. Dis..

